# Cytology history preceding cervical cancer diagnosis: a regional analysis of 286 cases

**DOI:** 10.1038/sj.bjc.6606067

**Published:** 2011-01-25

**Authors:** M Gök, L Rozendaal, J Berkhof, O Visser, C J L M Meijer, F J van Kemenade

**Affiliations:** 1 Department of Pathology, VU University Medical Center, Amsterdam 1007 MB, The Netherlands; 2 Department of Clinical Epidemiology and Biostatistics, VU University Medical Center, Amsterdam, The Netherlands; 3 Integraal Kankercentrum (Comprehensive Cancer Center), Amsterdam, The Netherlands

**Keywords:** population-based screening programme, non-compliance, cervical intraepithelial neoplasia, cervical carcinoma

## Abstract

**Background:**

Despite programmed screening in the Netherlands, the decrease in incidence of cervical carcinoma lags behind. We analysed screening results preceding carcinoma cases, timeliness in case of follow-up, and FIGO (International Federation of Gynaecology and Obstetrics) stages as efficiency parameters for screening were taken.

**Methods:**

We analysed 286 women with cervical cancer between 2005 and 2007 for cytology history preceding carcinoma, hierarchically arranging cytology history (if present) into three groups: ‘screened’, ‘work-up’, and ‘underscreened’ (>6 yrs before diagnosis). For screen- and work-up smears, we analysed timeliness. FIGO stage was measured in relation to cytology history.

**Results:**

A total of 105 out of 286 (36.7%) women with cervical carcinoma were screened preceding the diagnosis. Delayed time intervals in case of abnormal cytology were 43.5% for borderline/mild dyskaryosis (BMD) and 38.0% for BMD (moderate dyskaryosis or worse; *P*=0.51). A total of 108 out of 286 (36.4%) women were underscreened, and 73 out of 286 (25.5%) were unscreened. Advanced carcinoma or FIGO stage ⩾2B in screened women was 16.0 *vs* 48.7% in work-up, underscreened, or unscreened (*P*<0.001).

**Conclusion:**

Women with cervical cancer are underscreened and have poor timeliness in case of abnormal cytology. Being un- or underscreened correlates significantly with higher cervical cancer stages, especially in older women (aged ⩾49 years; *P*<0.001). Improvement of attendancy is needed to meet the standard of quality for screening programmes.

Cervical cancer is preceded by well-defined premalignant lesions, which can be identified by detecting abnormal cells in Papanicolaou smear. Cervical screening by cytology with adequate treatment have resulted in a decrease in incidence and mortality of cervical carcinoma (Gustafsson *et al*, 1997; Vizcaino *et al*, 2000). In the Dutch screening programme, women aged 30–60 years are invited every 5 years for seven times in a lifetime. Modelling, before the introduction of the Dutch cervical screening programme, predicted a decrease in cervical carcinoma by approximately 75%, assuming full coverage (van Ballegooijen, 1998) within the range mentioned in other studies (Sasieni and Adams, 1999; IARC, 2005).

Coverage of the screening programme is currently 77% (Rebolj *et al*, 2007). Approximately 65% of women attend the screening programme after an invitation, referred to as smears made inside the screening programme and 12% reflects smears made outside the screening programme (opportunistic smears). Approximately 23% of the invited women will not be screened at all (Bais *et al*, 2007). Collectively, the effect on carcinoma incidence through these two modes of screening will be lower than modelled for the programme, as full coverage is not attained. Moreover, the non-participating fraction of women (referred to as non-attendees) has a higher risk for cervical carcinoma than average, thus further decreasing the effectiveness of a programme in reducing carcinoma incidence (van Oortmarssen and Habbema, 1991). Earlier studies have shown that 40–50% of the women diagnosed with cervical cancer are in the non-compliance group (van der Graaf *et al*, 1986; Bos *et al*, 2006).

Here we analysed 286 women, with cervical carcinoma from the region Noord-Holland/Flevoland in the Netherlands, diagnosed between 2005 and 2007. We analysed the relationship between the FIGO (International Federation of Gynaecology and Obstetrics) stage of the detected carcinoma and the associated screen status. In addition, we analysed whether the smear was made within or outside the screening programme, and the compliance for referral to the gynaecologist in case of an abnormal smear.

## Materials and methods

### Data of regional carcinoma cases obtained from PALGA

All cytological and histological results carried out in the Netherlands are excerpted in the Pathological National Automated Archive (PALGA), a centralised database. Since 1991, coverage is at least 95% (Casparie *et al*, 2007). We linked patient records based on the identity of the encrypted first four letters of the maiden name and date of birth. The ‘twinning rate’ is estimated to be around 2% per record (Bos *et al*, 2002).

In total, our query in PALGA provided 337 830 smears of which 334 cases (0.10%) with index-diagnosis histologically confirmed cervical carcinoma in 2005–2007 and living in one region of the Netherlands. Group of records, presumably belonging to a single person, were ‘eyeballed’ (checking every case manually) to filter out administrative twins by checking domicile, initials, and apparent inconsistencies in clinical history (*n*=48). This left 307 298 numbers of smears of which 286 (0.09%) women were diagnosed with histologically confirmed cervical carcinoma. Cervical smears were registered as either inside- or outside the screening programme. This study was approved by the ethical committee of PALGA.

### Definition of ‘cytology history’ in this analysis

Screening histories of the 286 cases of carcinoma from PALGA database were analysed for presence or absence of cytology history. For each woman, we took time point with cervical carcinoma diagnosis (*t*=0). From this point, we did a retrospective research in the preceding time period to determine their last cytological examination. Three time frames were defined as follows: ‘unscreened’ (no smear at all before the diagnosis of cervical carcinoma or only work-up smear before the diagnosis), ‘underscreened’ (last smear taken >6 years before the diagnosis: the work-up smear not included), or ‘screened’ (smear taken between 1 and ⩽6 years before the diagnosis). We defined cytology obtained less than 1 year preceding the diagnosis as a ‘work-up smear’.

The choice of this time period is based on the ‘once in every 5-years invitation’ of the Dutch screening programme protocol, which means that all women between 30 and 60 years of age are invited for programmed screening for every 5 years, which is sent for free of charge for cytology analysis. In relation to this interval, we have defined in our analyses the screening episode with an interval period of 6 years before the diagnosis of cancer until 1 year before the diagnosis. If a smear is detected in the database within this period, we consider the women ‘screened’. Our three defined time frames were categorised hierarchically: first, a woman was considered ‘screened’ if she had a smear taken in this between 1 and ⩽6 year period. Second, a woman was considered ‘underscreened’ if she had a smear >6 years before the diagnosis, but not in the screened period. Third, a woman was considered ‘unscreened’ if she had only a work-up smear or had no smear at all in the past. Owing to this hierarchical categorisation, the women with a smear in the screened period could also have smears in the period >6 years before the diagnosis, and/or a work-up smear. Furthermore, women categorised as underscreened could also have work-up smear.

**Figure 1:**
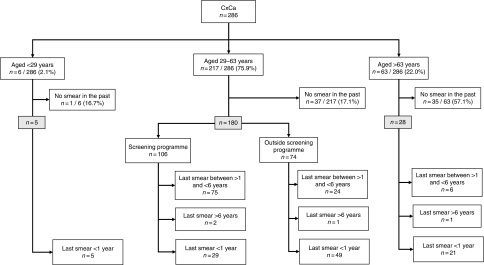
Flowchart of women with cervical carcinoma (stratified by three age groups, mode of screening, and cytology history). The flowchart is based on non-hierarchical categorisation of the cytology history.

Age stratification was carried out in seven groups of 5 years: 29–33, 34–38, 39–43, 44–48, 49–53, 54–58, 59–63 years of age; in addition, <29 years and >63 years of age. Sub-analysis of smears was based on the mode (i.e., invitational or inside the screening programme or outside the screening programme). For this sub-analysis of ‘within the screening programme’, we have chosen the upper age as 63 years, as the Dutch screening programme invites women up to age 60 years (with a cutoff at 63 years because of a possible follow-up time) and for lower age as 29 years, as women can be invited from the age of 29 years onwards. The number of eligible women (29–63 years) was 217 for this sub-analysis with respect to the the screening programme (Figure 1).

In case of multiple abnormal smears in the screening history (e.g., in case of repeat cytology after BMD), we accepted the first abnormal smear as the starting point. If women had only multiple normal smear results in the screened period, we analysed the time interval between the last smear before the diagnosis and at the time of diagnosis. All analyses were performed using SPSS 15.0 software (IBM Company, Armonk, NY, USA).

### Definition of work-up smear in this analysis

Work-up smears for diagnosis were defined as all cases of cytology obtained <1 year before the diagnosis rather than 6 months, because we considered women with a ⩾BMD preceded by BMD cytology in the period from 12 to 6 months before the diagnosis to represent women who should have had a severe abnormality in the first smear, and thus be representative for women with signs and symptoms of carcinoma. We have taken this into account plus allowing a few months delay in repeat. Thus, we end the period for work-up smears at 1 year before the diagnosis.

### Screening programme in the Netherlands and eligibility

Women are invited in the Netherlands into the programme in the year they turn 30 years of age. Actually, at the time of screening they may still be 29 years. Similarly, at the second invitation, women may be still 34 years of age.

Women with a normal smear results will be invited again for the next screening round. Women with borderline/mild dyskaryosis (BMD) are advised to repeat the smear after 6 and 18 months. If at least one of the repeated cytology smears is read as BMD or worse, the woman will be referred to a gynaecologist for colposcopy. Women with >BMD (moderate dyskaryosis or worse) are referred immediately to a gynaecologist. Women with inadequate smears (not suitable for diagnosis) are advised to repeat the test after 6 weeks.

### Time interval between abnormal smear and diagnosis of cervical carcinoma

Among the women with cervical carcinoma, we analysed the time interval between the first abnormal smear cytology and the histologically confirmed diagnosis. For timeliness, women were categorised as ‘not delayed’ or ‘delay in diagnosis’ (see Table 1 for definitions of time frames). Smears with BMD that led to the histologically confirmed diagnosis ⩽24 months were considered as ‘not delayed’. For smears with >BMD, timeliness was set at ⩽6 months as described by Bos *et al* (2006). For time-interval computations, all women with a smear taken up to 6 years (−2192 days) before the diagnosis were included in these analyses (thus encompassing both the screened group as the work-up group, *n*=195). Two cases with inadequate smears without follow-up were excluded, leaving a total of 193 cases. After tabulating smear results, the time interval was categorised in two periods that is, not delayed and delay in diagnosis.

### FIGO stage of cervical carcinoma related to cytology history and mode of screening

**Figure 2:**
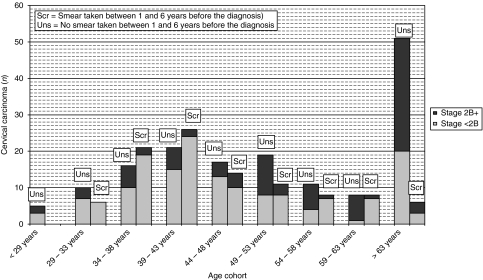
Women with histologically confirmed cervical carcinoma; the FIGO stage in relation to their screen smear, stratified by age cohort. Each age cohort contains two columns out of which the left column presents women without a smear in the screened period (1 and ⩽6 years preceding diagnosis), and the right column presents smear taken within this period.

The FIGO stage of cervical carcinoma was related to the cytology history (i.e., presence or absence of a smear). Absence was defined as having no smear in the screened period. Furthermore, we also analysed the FIGO stage in relation to the cytology result of women with a smear taken in the screened period, allowing insight in whether an inadequate cytology advice results in a later detection of carcinoma. Both types of analyses were stratified by age eligibility for screening invitation, or other ages. These analyses were also stratified by age (Figure 2). The analyses were carried out with the Fisher's exact test.

## Results

### Cases of cervical carcinoma

The final query result from PALGA for the period from 2005 to 2007 provided 286 cases after excluding 48 cases because of double-counting twinning, too late a diagnosis (i.e., in 2008), carcinoma of endometrial origin or metastasis. Age ranged between 25 and 93 years old (mean age, 50.8 years). We divided the remaining 286 women into an eligible group (*n*=217, aged 29–63) for receiving an invitation within the context of a screening programme and a non-eligible group (i.e., aged <29 (*n*=6), or >63 years (*n*=63) for falling outside invitational cohorts. The number of women without any smear in the period preceding the diagnosis was 1 out of 6 (16.7%), 37 out of 217 (17.1%), and 35 out of 63 (55.6%) for women aged 29, 29–63, and >63 years, respectively (*n*=217; Figure 1).

### Cytology preceding carcinoma cases: un(der)screened, screened, or work-up smears

The hierarchically categorised Table 2 shows that the number of women in the screened period is 105 out of 286 (36.7%). In addition, 18 out of 286 women (6.3%) were underscreened (i.e., no smear between 1 and ⩽6 years). Furthermore, 90 out of 286 women (31.5%) only had a work-up smear, and 73 out of 286 women (25.5%) had no smear at all before the histologically confirmed diagnosis. The latter two sets represent the group of unscreened women. As can be seen in the one, but in the lowest row of Table 2, a sub-analysis for women eligible for screening programme invitation, showed that 180 out of 217 (99+16+65) women (82.9%) had at least a smear anytime preceding the diagnosis. This percentage was composed of 45.6% for ‘screened’, and 37.3% (7.4%+30.0%) for ‘underscreened’ and work-up smear. A total of 37 out of 217 (17.1%) were ‘unscreened’, and had also no work-up smear before the diagnosis.

### Cytology results in relation to cytology history

Table 3 shows the cytology result of historical smears. Interestingly, 69 of 105 women (63.8%) who were screened between 1 and ⩽6 years had a normal or inadequate (*n*=2) cytology, respectively. Furthermore, 15 out of 105 (14.3%) had >BMD, but cervical cancer was diagnosed more than a year later, suggestive of time delays. For women with only a work-up smear preceding the diagnosis, the results show a different pattern: >BMD was found in 75 out of 90 women (83.3%). The results of cytology in the group of women screened >6 years before the diagnosis resembled the first group: 12 out of 18 women (66.7%) had a normal cytology result, 5 out of 18 women (27.8%) had BMD cytology result, and only 1 out of 18 (5.6%) had >BMD (Table 3).

In addition, we have sub-divided our group of underscreened women (*n*=18), with respect to the time interval between the last negative smear and the diagnosis of carcinoma, into a subgroup with the last smear made 7–11 years previously for diagnosis and a group with the last smear made ⩾11 years before diagnosis. We could not find a significant difference in cytology result. However, it should be realised that the number of women in the subgroups was very small for a meaningful analysis (data not shown).

### Diagnosis of work-up smear in women with normal cytology results in the screened period

The normal (*n*=67) and inadequate (*n*=2) cytology results of women in the screened period, as shown in Table 3, does not exclude the possibility that these women had a work-up smear as well (as our analysis is hierarchical). Of the 69 women, 41 subsequently had >BMD (including the two women with inadequate cytology smear; 59.4%) and 9 had BMD (13.0%) as work-up smear. Furthermore, 2 out of 69 (2.9%) and 1 out of 69 (1.4%) had normal cytology or inadequate cytology, respectively. The remaining 16 out of 69 women (23.2%) had no work-up smear. When further dividing these 53 work-up smears by the mode of screening (‘within screening programme’ and ‘outside screening programme’), the mode was not significantly different: 26 out of 53 women (49.1%) with a work-up smear were found within screening programme *vs* 27 out of 53 (50.9%) outside the programme.

In addition, we analysed possible work-up smears from women with >BMD and BMD in the screened period. A total of 12 out of 15 women (80.0%) with >BMD in the screened period again had >BMD, one woman (6.7%) had BMD, and two women (13.3%) had no smear in the work-up for diagnosis period (not shown). Similarly, for women with BMD in the screened period, 13 out of 21 (61.9%) had >BMD, 3 out of 21 (14.3%) had BMD, 1 woman (4.8%) had a normal cytology result, and 4 women (19.0%) had no smear in the work-up period. Again, the distribution of the indication smear is equal for both the groups (data not shown).

### Screen and work-up smears in women with carcinoma in relation to mode of screening

For the sub-analysis of cytology history in relation to the mode of screening (stratified by age cohort), only women eligible (*n*=217) for programmed screening (aged 29–63 years) and having at least one smear ⩽6 years before diagnosis (either screen smear or work-up smear; for definitions see Table 1) were selected (Table 4). Screen and work-up smears are denoted separately. In addition, 14 out of 18 women (77.8%) who were categorised as underscreened also had a work-up smear and were therefore included. The remaining four women from this group were added to the 73 women without any smear before the diagnosis, as a work-up smear was lacking.

Only 99 of 217 women (45.6%) actually had a smear taken in the screened period (*P*<0.001), of whom 75 out of 99 (75.8%) had a programmed smear and 24 out of 99 (24.2%) had the smear taken outside the screening programme, which was statistically significant (*P*<0.001).

A further 78 out of 217 (35.9%) women had a work-up smear only, divided between 29 out of 78 (37.2%) (aged 29–63 years) for invitational cytology and 49 out of 78 (62.8%) outside the screening programme (Table 4 and Figure 1). In total, 40 out of 217 (18.4%) women had no smear at all ⩽6 years before the diagnosis.

### Timeliness between first abnormal cytology smear and histologically confirmed carcinoma

Table 5 shows the timeliness between the first abnormal smear and histologically confirmed cervical cancer between 2005 and 2007 (stratified by cytology result: >BMD or BMD). We included all cytology analysis results taken up to 6 years before the diagnosis (thus both screen smear as well as work-up smear), which resulted in total 142 >BMD and 46 BMD cases. As shown previously, Table 3 shows that 15 and 21 women with cytology in the screened period had >BMD and BMD, respectively. Furthermore, cases with ⩾BMD work-up smear of women, who had a normal or inadequate cytology smear in the screened period before diagnosis, were included (41 and 9 out of 69 women had >BMD and BMD, respectively).

For timeliness, we divided the interval as ‘not delayed’ and ‘delay in diagnosis’ (see Materials and methods for definitions). In the group of women with >BMD cytology smear, 88 out of 142 (62.0% 95% CI: 53.5–70.0) showed no delay for diagnosis. In the group, women with BMD, 26 out of 46 (56.5% 95% CI: 41.1–71.1) had no delay in diagnosis. Delay in diagnosis for women with >BMD and BMD were observed in 54 out of 142 (38.0%) and 20 out of 46 (43.5%) women, respectively. Overall, 60.6% of the women had no delay in diagnosis (95% CI: 53.3–67.7). The difference in delay in diagnosis of between women with >BMD and BMD cytology smears was not significantly lower (*P*=0.51).

We also analysed the work-up smears of women who had a normal smear (12 out of 18 women; 66.7%) in the period >6 years before the diagnosis. In total, 10 of these 12 women had >BMD (83.3%) and 2 out of 12 women (16.7%) had again normal cytology result. A total of 6 out of 18 women with ⩾BMD result (one woman with >BMD and five women with BMD) >6 years before diagnosis were categorised as delay in diagnosis. Together, with the 75 women with >BMD and 11 women with BMD, the total >BMD and BMD cases are 142 and 46, respectively. For the remaining 25 women, timeliness was not applicable, as they had had either normal cytology (*n*=23) or time information was lacking (*n*=2).

### FIGO stage of cervical carcinoma in relation to cytology history

In Table 6a, we show the FIGO stage in relation to the cytology history. The FIGO stage was grouped into low (1A or 1B), borderline (2A–2B), and high grades (⩾3A) because this grouping has consequences for therapy, and gives more relevant information about the relation between FIGO stage and screening history. FIGO stage information was available for 90.2% (258 out of 286) of the cases, of which 196 and 62 in the group of women aged 29–63 and <29 and/or >63 years, respectively. Most women had no cytology smear taken in the screened period (158 out of 258; 61.2%, 95% CI: 55.3–67.2%), leaving 100 women with a smear taken in the screened period with a known FIGO stage in this group for analyses. In total, 18 out of 34 women (52.9%) with FIGO stage 1A had screen smear. Furthermore, 58 out of 95 (61.1%), 15 out of 42 (35.7%), and 3 out of 25 women (12.0%) with a screen smear had 1B, 2A–2B, and 3A–4 FIGO stages, respectively.

When comparing the FIGO stage 1A with 1B, there is statistically no difference (*P*-value, 0.424) in the percentage of screen smear. However, when we compare FIGO stage group 1B with 2A–2B, we notice a significant difference (*P*-value, 0.009). Also a difference can be noticed when we compare the group 2A–2B with 3A–4 (*P*-value, 0.047).

Overall, the group with high-grade FIGO stage (2A or higher) had significantly lower screen smear compared with the group with low-grade FIGO stage (1A or 1B) (*P*<0.001; Table 6a). In the age groups <29 and/or >63 years, there were no significant differences between the FIGO stage and having had a screen smear (*P*=0.20). Furthermore, we also analysed whether the differences between FIGO stage and two age groups (29–63 *vs* <29 and/or >63 years) were significant. The result showed a significant value at *P*<0.001 (*P*-value not shown in Table 6a).

When we analysed FIGO stage of women with a screen smear (*n*=100) in relation to their cytology smear result (>BMD, BMD, or normal) no significant differences were found (Table 6b). However, women aged 29 and/or >63 years showed significantly more cytology lesions than women aged 29–63 years (*P*<0.001; data not shown).

We then analysed FIGO stage in relation to screen smear *vs* otherwise per-age strata (Figure 2). We noticed a trend of more severe FIGO stage (FIGO stage ⩾2B) among older women (aged 49 years and older; *n*=56 out of 89; 62.9%) who had no smear in the screened period, compared with women who had their smear in the same period (*n*=8 out of 33; 24.2%, *P*<0.001). In addition, we analysed whether a correlation could be found between the mode of screening and severe FIGO stage found among women (aged 34–63 years) who had their smear taken in the screened period. No statistical difference was found (*P*=0.822; data not shown).

## Discussion

Our data show that only 36.7% (105 out of 286) of women diagnosed with cervical carcinoma in the region of Noord-Holland/Flevoland between 2005 and 2007 were appropriately screened. If restricted to eligible women for receiving an invitation for cervical screening (aged 29–63 years), this percentage was 45.6% (99 out of 217). This finding is in agreement with a meta-analysis study, showing that about 46.2% women had a smear in the screened period (Spence *et al*, 2007).

Further analysis showed that 67 out of 105 women (63.8%) that were ‘screened’ had a normal cytology result (Table 3). A meta-analysis study had an outcome of 29.3% of women with at least one normal cytology result in the same period (Spence *et al*, 2007). However, 48 out of the 67 (74.5%) women in our study subsequently had abnormal cytology (⩾BMD) in their work-up smear, as assumed in previous modelling. Only two women (3.9%) had again a normal smear result in the work-up smear period. Even within the context of the limited reproducibility of cytology, this change from normal cytology to abnormal cytology in women with subsequent histologically proven cancer is likely to be because of inappropriate sampling, processing, or erroneous reading of the cytology.

Furthermore, our data show that 75 out of 217 (34.5%) women from the eligible age cohorts had been screened within the screening programme (Table 4) in the appropriate period preceding the carcinoma diagnosis. From the residual of 142 patients, 29 were possibly ‘screen detected’ within the programme (13.4%) but were underscreened. The remaining 113 out of 217 eligible women, either did not have cytology ⩽6 years preceding diagnosis (underscreened period; 40 out of 217; 18.4%), or had obtained a smear outside the programme (73 out of 217; 33.6%). These data show that the detected (histologically confirmed) carcinoma rate was higher among women who had not sufficiently attended the organised screening (142 out of 217) in the screened period (65.4 *vs* 34.6%. *P*<0.001; Table 4).

The result of timeliness between the first abnormal smear ⩽6 years, preceding the diagnosis and histologically confirmed diagnosis, showed in 62.0% (95% CI: 53.5–70.0%) no delay in diagnosis among women with >BMD as cytology diagnosis and in 56.5% (95% CI: 41.1–71.1%) among women with BMD (*P*=0.51). This suggests that significant improvement can be made in both the compliances with repeats as well as re-dressing delays in workup.

The correlation between the FIGO stage and women with or without a cytology smear in screened period showed a difference for higher stages: a 1.7-fold higher cervical carcinoma stage (stages 2A–2B) was observed in carcinoma-bearing women (aged 29–63 years) without cytology history compared with women at 1B FIGO stage (*P*=0.009), whereas 1.4-fold higher cervical carcinoma stage (stages 3A–4) was found among women without screen smear compared with women with screen smear and at 2A–2B FIGO stage (*P*=0.047). Furthermore, a trend can be seen in the relation between the higher stages and age of women. The older women (age ⩾49 years) without a smear in the screened period showed significantly higher FIGO stages compared with older women who had a smear in the same period (*P*<0.001). This indicates that non-screened older women are at higher risk for high-stage cervical cancer.

In conclusion, our data show that only 34.6% of all eligible women (aged 29–63 years) had participated in an organised programmed screening between 1 and ⩽6 years before diagnosis (95% CI: 28.2–40.9% *P*<0.001). Even if women screened outside the screening programme were included, the percentage would be 45.6%, which is similar to as reported in the literature (Spence *et al*, 2007). Moreover, 67 women had a normal screen smear, followed by an abnormal smear in either the next round or in the work-up phase before carcinoma. These findings demonstrate both programme sensitivity as well as compliance needs improvement. This may be achieved by implementing hrHPV testing as the primary screening tool (Cuzick *et al*, 2006; Bulkmans *et al*, 2007; Mayrand *et al*, 2007; Ronco *et al*, 2008, 2010).

There are several options to improve the participation rate in organised screening programme. Hermens *et al* (2000) have shown that women are more willing to attend a screening programme when invitations are sent by their general practitioners instead by their municipality. Another option to influence the effectiveness of the screening programme, especially with regard to timeliness, is increased computerised controls to support physicians in controlling follow-up (Hermens *et al*, 1999). Finally, we recently showed that offering self-sampling for hrHPV DNA testing to non-attendees in the cervical screening programme is a feasible and effective approach, leading both to increased coverage and marked detection of CIN2^+^/CIN3^+^ lesions, particularly in women who did not attend the previous round of screening (Gok *et al*, 2010).

## Figures and Tables

**Table 1:** Overview of the definitions in this manuscript: screen smear (screening episode), work-up smear, and delay in diagnosis and screening interval

**Definition**	**Description**
Screen smear	Smear taken between 1 and ⩽6 years preceding the diagnosis
Work-up smear	Smear taken maximum at 1 year preceding the diagnosis
Screening interval in Dutch programme	Once in every 5 years (between 30 and 60 years)
	
**‘Delay’ in diagnosis**	**Interval between last smear and diagnosis**
Delay for BMD	Cytology>18 months preceding the diagnosis
Delay for worse than BMD (>BMD)	Cytology>6 months preceding the diagnosis

Abbreviation: BMD=borderline/mild dyskaryosis.

**Table 2:** The cytology history (hierarchical categorised into smear taken between 1 and 6 years, smear >6 years, and <1 year or no smear) of women with histologically confirmed cervical cancer diagnosis between 2005 and 2007, stratified by age cohort

	**Screen smear (i.e., between 1 and 6 years)**	**Smear taken** >**6 years**	**Smear taken <1 year** a	**No smear**	**Total**
**Age cohort**	***N* (% of row totals)**	***N* (% of row totals)**	***N* (% of row totals)**	***N* (% of row totals**)	***N* (% of row totals)**
⩽28 years	—	—	5 (83.3)	1 (16.7)	6 (2.1)
29–33 years	6 (30.0)	—	11 (55.0)	3 (15.0)	20 (7.0)
34–38 years	23 (56.1)	—	12 (29.3)	6 (14.6)	41 (14.3)
39–43 years	28 (54.9)	5 (9.8)	10 (19.6)	8 (15.7)	51 (17.8)
44–48 years	14 (43.8)	3 (9.4)	11 (34.4)	4 (12.5)	32 (11.2)
49–53 years	11 (32.4)	3 (8.8)	11 (32.3)	9 (26.5)	34 (11.9)
54–58 years	9 (42.9)	4 (19.0)	6 (28.6)	2 (9.5)	21 (7.3)
59–63 years	8 (44.4)	1 (5.6)	4 (22.2)	5 (27.8)	18 (6.3)
⩾64 years	6 (9.5)	2 (3.2)	20 (31.7)	35 (55.6)	63 (22.0)
Total (aged 29–63 years)^b^	99 (45.6)	16 (7.4)	65 (30.0)	37 (17.1)	217 (100)
Total	105 (36.7)	18 (6.3)	90 (31.5)	73 (25.5)	286 (100)

a Smear taken <1 year before the diagnosis is considered as work-up smear for diagnosis.

b Women ageing 29–63 years with non-attendancy possibility in the previous programmed screening round.

**Table 3:** Screen smears, smears taken more than 6 years ago, and work-up smears in women with histologically confirmed cervical cancer diagnosis arranged according to cytology results

	**Screen smear (i.e., between 1 and 6 years)**	**Smear taken** >**6 years**	**Work-up smear (<1 year)^a^**	**No smear at all**	**Total**
**Cytology**	**Number (% of column totals)**	**Number (% of column totals)**	**Number (% of column totals)**	**Number (% of column totals)**	**Number (% of column total)**
>BMD	15 (14.3)	1 (5.6)	75 (83.3)	N/A	91 (31.8)
BMD	21 (20)	5 (27.8)	11 (12.2)	N/A	37 (12.9)
Normal	67 (63.8)	12 (66.7)	2 (2.2)	N/A	81 (28.3)
Inadequate	2 (1.9)	—	2 (2.2)	N/A	4 (1.4)
No smear	N/A	N/A	N/A	73 (100)	73 (25.5)
Total	105 (100)	18 (100)	90 (100)	73 (100)	286 (100)

Abbreviations: BMD=borderline/mild dyskaryosis; N/A=not available.

a Smear taken <1 year before the diagnosis.

**Table 4:** Screening mode of screen smear (i.e., between 1 and 6 years before the diagnosis) or work-up smear (<1 year before the diagnosis) sub-divided into age cohorts of all 286 cervical carcinoma cases

	**Screen smear (i.e., between 1 and 6 years before the diagnosis)**		**Work- up smear (i.e., <1 year before the diagnosis)^a^**			
	**Screening programme**	**Outside screening programme**	**Screening programme**	**Outside screening programme**	**Cytology** >**6 years or no cytology preceding the diagnosis**	**Total**
	**Number (% of row totals)**		**Number (% of row totals)**		**Number (% of row total)**	**Number (% of column total)**
⩽28 years	—	—	—	5 (83.3)	1 (16.7)	6 (2.1)
Age 29–33 years	5 (25.0)	1 (5.0)	8 (40.0)	3 (15.0)	3 (15.0)	20 (7.0)
Age 34–38 years	19 (46.3)	4 (9.8)	2 (4.9)	10 (24.4)	6 (14.6)	41 (14.3)
Age 39–43 years	19 (37.3)	9 (17.6)	8 (15.7)	6 (11.8)	9 (17.6)	51 (17.8)
Age 44–48 years	11 (34.3)	3 (9.4)	3 (9.4)	11 (34.4)	5 (15.6)	32 (11.2)
Age 49–53 years	7 (20.6)	4 (11.8)	3 (8.8)	11 (32.4)	9 (26.5)	34 (11.9)
Age 54–58 years	6 (28.6)	3 (14.3)	2 (19.0)	6 (28.6)	4 (19.0)	21 (7.3)
Age 59–63 years	8 (44.4)	—	3 (16.7)	2 (11.1)	5 (27.8)	18 (6.3)
⩾64 years	2 (3.2)	4 (6.3)	—	21 (33.3)	36 (57.1)	63 (22.0)
Total screening age (29–63 years)	75 (34.6)	24 (11.1)	29 (13.4)	49 (22.6)	40 (18.4)	217 (100)
Total overall	77 (26.9)	28 (9.8)	29 (10.1)	75 (26.2)	77 (26.9)	286 (100)

The cases of women within age cohorts (29–63 years; *n*=217) of the Dutch screening programme indicated as age strata.

a This includes women with a work-up smear, who also had a smear taken >6 years before the diagnosis but not between ⩽1 and 6 years before the diagnosis: 14 out of 18 (77.8%) women who were categorised in the period >6 years before the diagnosis had a work-up smear. The remaining four women are included in the category No cytology <6 years before the diagnosis, along with women without any smear in the past (*n*=73), giving a total of 77.

**Table 5:** Timeliness of abnormal (‘>BMD’ or ‘BMD’) and normal smear preceding cervical cancer. All smear types (smear and work-up smears)

**Cytology**	**Total, *n* (%)**	**Not delayed, *n* (%)**	**95% CI**
>BMD^a^	142 (100)	88 (62.0)	53.5–70.0
BMD^b^	46 (100)	26 (56.5)	41.1–71.1
Total	188^c^ (100)	114 (60.6)	53.3–67.7

Abbreviations: BMD=borderline/mild dyskaryosis; CI=confidence interval.

a Maximum time interval between the first abnormal smear and histologically confirmed CxCa was 6 months (a longer time interval was considered as delay in the diagnosis).

b Maximum time interval between the first abnormal smear and histologically confirmed CxCa was 24 months (a longer time interval was considered as delay in the diagnosis).

c In total, 73 out of 286 women had no smear at all preceding the diagnosis, thus leaving 213 cases with a smear. A total of 25 out of these 213 women had only normal cytology (*n*=23) or information on time was lacking (*n*=2), leaving 188 cases to analyse. This group consisted of >BMD or BMD as follows: in the screen period 15 and 21 women had >BMD and BMD, respectively (Table 3). In the work-up period group, 75 and 11 women had >BMD and BMD, respectively. Furthermore, 41 and 9 women who had a normal smear result in the screen period had >BMD and BMD, respectively, as work-up smear. Finally, for women who had a normal smear result in the underscreened period (*n*=12), 10 women had >BMD as work-up smear (The remaining two woman had normal smear result again). Another one and five women had already >BMD and BMD, respectively, in the underscreened period (Table 3). This results in a total of 142 >BMD and 46 BMD women (total *n*=188).

**Table 6a:** Women with a smear *vs* women without a smear in screen smear period, stratified according to FIGO stages, and eligibility for screening programme (aged 29–60 years, or otherwise)

	**Cervical carcinoma**	**Screen smear (i.e., between 1 and 6 years)^a^**		**Odds ratio**	
**FIGO stage**	**Number**	**Number**	**Percentage within stage (95% CI)**	**Estimate (95% CI)**	***P*-value**
*Aged 29–63 years*					
Stage 1A	34	18	52.9 (35.1–70.2)	1.39 (0.58–3.30)	0.424 (stage 1A *vs* 1B)
Stage 1B	95	58	61.1 (50.5–70.9)	0.35 (0.15–0.80)	0.009 (stage 1B *vs* 2A–2B)
Stages 2A–2B	42	15	35.7 (21.6–52.0)	0.24 (0.41–1.05)	0.047 (stages 2A–2B *vs* 3A–4)
Stages 3A–4	25	3	12.0 (2.5–31.2)	—	
Stage unknown	21	5	23.8 (8.2–47.2)	—	
Total	217	99	45.6 (38.9–52.5)		<0.001
					
*Aged* <*29 or* >*63 years*					
Stage 1A	5	1	20.0 (0.5–71.6)	1.00 (0.39–73.56)	
Stage 1B	10	2	20.0 (2.5–55.6)	0.57 (0.55–8.18)	
Stages 2A–2B	24	3	12.5 (2.7–32.4)	—	
Stages 3A–4	23	—	—	—	
Stage unknown	7	—	—	—	
Total	69	6	8.7 (3.3–18.0)		0.211

Abbreviations: BMD=borderline/mild dyskaryosis; CI= confidence interval; FIGO=International Federation of Gynaecology and Obstetrics.

a Include smears taken ⩽1 year before the diagnosis, smears taken >6 years before the diagnosis, and women who never had a smear taken before the diagnosis.

**Table 6b:** Results of screen smear stratified according to FIGO stage

	>**BMD**	**BMD**	**Normal**	**Inadequate**	**Total**	
**FIGO stage**	**Number (% of column total)**				**Number (% of column total)**	***P*-value**
*Aged 29–63 years*						
Stage 1A	12 (36.4)	10 (30.3)	11 (33.3)	—	33 (18.3)	0.151
Stage 1B	26 (30.2)	15 (17.4)	44 (51.2)	1 (1.2)	86 (47.8)	0.620
Stages 2A–2B	11 (39.3)	4 14.3)	12 (42.9)	1 (3.6)	28 (15.6)	0.346
Stages 3A–4	4 (25.0)	5 (31.3)	6 (37.5)	1 (6.3)	16 (8.9)	
Stage unknown	13 (76.5)	1 (5.9)	3 (17.6)	—	17 (9.4)	
Total	66 (36.7)	35 (19.4)	76 (42.2)	3 (1.7)	180 (100)	
						
*Aged* <*29 or* >*63 years*						
Stage 1A	2 (66.7)	—	1 (33.3)	—	3 (9.1)	
Stage 1B	5 (71.4)	2 (28.6)	—	—	7 (21.2)	
Stages 2A–2B	9 (69.2)	—	4 (30.8)	—	13 (39.4)	
Stages 3A–4	4 (100)	—	—	—	4 (12.1)	
Stage unknown	5 (83.3)	—	—	1 (16.7)	6 (18.2)	
Total	25 (75.8)	2 (6.1)	5 (15.2)	1 (3.0)	33 (100)	

Abbreviations: BMD=borderline/mild dyskaryosis; FIGO=International Federation of Gynaecology and Obstetrics.

Of the 217 total cases in the age group 29–63 years, 37 of the women were without any smear, leaving 180 cases behind. In 17 cases, FIGO stage could not be assessed. Of the 69 women, in age groups <29 and/or >63 years, 36 were without any preceding smear, leaving 33 cases. In six cases, no FIGO assessment was available.
